# New Marginal Spectrum Feature Information Views of Humpback Whale Vocalization Signals Using the EMD Analysis Methods

**DOI:** 10.3390/s23167228

**Published:** 2023-08-17

**Authors:** Chin-Feng Lin, Bing-Run Wu, Shun-Hsyung Chang, Ivan A. Parinov, Sergey Shevtsov

**Affiliations:** 1 Department of Electrical Engineering, National Taiwan Ocean University, Keelung 20224, Taiwan; pooukimo@hotmail.com; 2 Department of Microelectronics Engineering, National Kaohsiung University of Science and Technology, Kaohsiung 81157, Taiwan; 3 I. I. Vorovich Mathematics, Mechanics, and Computer Science Institute, Southern Federal University, 344090 Rostov-on-Don, Russia; iparinov@sfedu.ru; 4 Head of Aircraft Systems and Technologies Laboratory, South Center of Russian Academy of Science, 344006 Rostov-on-Don, Russia; shevtsov@ssc-ras.ru

**Keywords:** humpback whale vocalization, intrinsic mode function, marginal spectrum, feature information

## Abstract

Marginal spectrum (MS) feature information of humpback whale vocalization (HWV) signals is an interesting and significant research topic. Empirical mode decomposition (EMD) is a powerful time–frequency analysis tool for marine mammal vocalizations. In this paper, new MS feature innovation information of HWV signals was extracted using the EMD analysis method. Thirty-six HWV samples with a time duration of 17.2 ms were classified into Classes I, II, and III, which consisted of 15, 5, and 16 samples, respectively. The following ratios were evaluated: the average energy ratios of the 1 first intrinsic mode function (IMF1) and residual function (RF) to the referred total energy for the Class I samples; the average energy ratios of the IMF1, 2nd IMF (IMF2), and RF to the referred total energy for the Class II samples; the average energy ratios of the IMF1, 6th IMF (IMF6), and RF to the referred total energy for the Class III samples. These average energy ratios were all more than 10%. The average energy ratios of IMF1 to the referred total energy were 9.825%, 13.790%, 4.938%, 3.977%, and 3.32% in the 2980–3725, 3725–4470, 4470–5215, 10,430–11,175, and 11,175–11,920 Hz bands, respectively, in the Class I samples; 14.675% and 4.910% in the 745–1490 and 1490–2235 Hz bands, respectively, in the Class II samples; 12.0640%, 6.8850%, and 4.1040% in the 2980–3725, 3725–4470, and 11,175–11,920 Hz bands, respectively, in the Class III samples. The results of this study provide a better understanding, high resolution, and new innovative views on the information obtained from the MS features of the HWV signals.

## 1. Introduction

### 1.1. The Related References

Hilbert–Huang transformation (HHT) time–frequency (TF) analysis is an interesting research topic. It is an empirical method that can be used to analyze non-stationary and non-linear signals [1,2]. Moreover, it is an adaptive signal-analysis scheme, which implies that the definition of the basis is signal-dependent. Further, the HHT-TF analysis scheme has revealed physical concepts in several instances of signal analyses. The aim of signal TF analysis is to extract information from the signal to demonstrate the underlying mechanisms, structures, and actual behaviors of various physical phenomena [3]. The non-stationary and non-linear characteristics of a signal with short durations can be extracted using the HHT technique. The original motivation for HHT technique development was to determine a technique that could provide insight into hydrospheric conditions by observing ocean topography. HHT was proposed as the solution for the non-linear class of spectrum analysis problems [4]. Empirical mode decomposition (EMD) followed by the Hilbert transform (HT) of the empirical decomposition data can be used for the analysis of non-linear and non-stationary data using engineering a posteriori data processing based on the EMD algorithm. This results in a non-constrained decomposition of a source real value data vector into a finite set of intrinsic mode functions (IMFs) and one residual function (RF) that can be further analyzed for the TF signal interpretation by the classical HT. The EMD-based Hilbert spectral analysis method with application to the non-linear wave evolution processes, the spectral form of the random wave field, and turbulence were studied [5]. The HHT method provides not only a more precise definition of particular events in the TF space but also more physically meaningful interpretations and new views of the underlying non-linear and non-stationary dynamic processes.

Adam [6,7,8] demonstrated the concept of HHT-TF-based analysis of marine mammal signals, and an analytical scheme for sperm and killer whale (KW) vocalizations was presented. KW vocalization with a time duration of 650 ms was analyzed using HHT methods. Thirteen IMFs and one RF were adaptively decomposed using the EMD method [6]. Accordingly, denoising and feature extraction can be achieved for KW vocalization. One hundred regular clicks of the same single sperm whale (SW) with a time duration of 14 ms were adaptively decomposed into nine IMFs and one RF using the HHT method [7]. Subsequently, denoising SW clicks was investigated. Lin et al. [9] proposed an advanced HHT-based approach to demonstrate the energy–frequency distributions corresponding to the click signals of SWs. The spatial energy–frequency characteristics of IMFs, and one RF for the click I and II samples were explored. Wen et al. [10] extracted the energy characteristics distributions of IMFs and one RF using EMD schemes to determine the B call vocalizations of blue whales (BWs). The detection schemes of the B call vocalizations of BWs were proposed using EMD-based energy spectrum entropy distribution [11].

The Navy’s highly secret sound surveillance system, which consists of arrays of bottom-mounted hydrophones, was developed to detect, localize, and track Soviet submarines during the Cold War. In 1993, the Comprehensive Nuclear-Test-Ban Treaty Organization was proposed, and a system comprising 11 hydroacoustic stations with bottom-mounted sensors was developed to detect seismic and acoustic waves owing to nuclear detonation at any location worldwide. Many of the current passive acoustic monitoring systems (PAMS) have evolved from the long-term monitoring system of bottom-mounted low-frequency seismic sensors owing to research by geophysicists. These PAMS are able to detect blue and fin whales (BFW) that emit very low-frequency sounds (LFS) between 10 and 20 Hz [12]. Goold et al. [13] analyzed the distinctive peak (DP) in the spectra of bull male sperm whale vocalizations (BMSWV) using Fourier analysis, and the results showed that the frequency range was 400 Hz–2 kHz. Keating et al. [14] analyzed the echolocation signals (ES) of Blainville’s beaked whales (BBW) using frequency-modulated pulses. The center frequency, 10 dB bandwidth, duration, and inter-pulse interval for the echolocation signals were 39.5 kHz, 9.3 kHz, 78 μs, and 209 ms, respectively. Houser et al. [15] investigated frequency-modulated tonal up-chirp (FMTUC) stimuli which may enhance auditory brainstem response amplitudes in KWs. The duration of the FMTUC could reach 1400 μs, whereas its spectral density was flat (±4 dB) across the stimulus bandwidth (10–130 kHz). The detections of Balaenoptera omurai whale vocalizations (BOWV) in soundscape recordings with a frequency range of 15–62 Hz were compared using spectrogram cross-correlation, entropy computation, and spectral intensity computation [16]. The time taken for analysis was ≤15 s. The high and low-frequency components of biphonic vocalizations in resident-type (R-type) KWs are not yet clearly understood [17]. Biphonic vocalizations have two independently modulated frequency components and play an important role in pod communication. The possible physical meanings of biphonic vocalizations in R-type KWs were examined in [18]. An autonomous recorder and a towed hydrophone array were deployed to record stereotyped down-swept contour vocalizations (DSCV) for KWs in the North Pacific. High-frequency modulated signals were recorded with a frequency range of 15.7–21.6 kHz, a 10 dB bandwidth of 5.9 kHz, and an analysis time of 65.2 ms. An earlier report used summed auto-correlation and Fourier transform frequency analysis methods to measure the pulse rate and peak frequency for Southeast Pacific blue whale (BW) song types [19]. The peak frequency of these s vocalizations was approximately 32 Hz.

Frazer et al. [20] found the frequency range of humpback whale vocalization (HWV) to be from 20 Hz to 8 kHz, whereas Au et al. [21] reported that the high-frequency harmonics of HWV songs extended beyond 24 kHz. Mature male HWVs produce elaborate acoustics in low-frequency bands of 0–1.5 kHz. Daily root-mean-squared sound pressure levels can be calculated to compare variations in low-frequency acoustic energy and monitor the population of HWV [22]. Male HWV presents long, structured sequences of acoustic vocalization, and the frequency distributions of the mean pulse-repetition rate can reach 3.97 kHz. Male HWV songs have a minimum frequency below 400 Hz and a maximum frequency above 3 kHz but below 8 kHz [23]. The songs are loud and of long duration; they are produced in the frequency range of 8 Hz–8 kHz, last from several minutes to hours, and have been noted to be frequency and amplitude modulated [24]. Angela et al. [25] examined the non-song vocalizations of HWV frequencies, which vary from 9 Hz to 6 kHz. The frequencies of the majority of vocalizations were under 200 Hz, and the duration of non-song vocalizations was found to be between 0.09 and 3.59 s.

Table 1 lists the comparison of features extracted from BFWV, BMSWV DP, BBW ES, KW FMTUC, BOWV, KW DSCV, BW song, HWVs, HWV, HWV songs, and HWV non-song. The Fourier method is often used to analyze HWVs. The energy in the frequency domain is a representation of the original HWVs. However, the Fourier method is not used to analyze nonlinearity and non-stationarity signals. The EMD-based analysis method [9] can be used to distribute the energy over the space–time–frequency space as a representation of the HWVs. Several IMFs and one RF signal (spaces) are generated in parallel and analyzed using the EMD method. The analysis method can be adopted to non-linearity and non-stationarity signals. It is suitable for extracting features from HWVs. The aims of this study are to provide a better understanding, high resolutions, and new perspectives regarding the MS features innovation information contained in HWV signals.

Zhang et al. [26] developed a 3D spatial and spectral-aware convolution module in which the spatial and spectral features of the target spectrum were extracted using 3D convolution. The spatial and texture features were extracted using a 2D convolution module with channel and spatial attention. A hyperspectral image dataset containing 1200 samples taken from ten corn varieties was constructed. The nondestructive identification of corn seeds was demonstrated using a hyperspectral image.

The remainder of this paper is organized as follows. Section 1.2 demonstrates the related EMD-based MS analysis method. Section 2 presents the humpback whale vocalization (HWV) samples. Section 3 presents the analysis results. The discussions and concluding remarks are presented in Section 4 and Section 5.

### 1.2. The Related EMD-Based MS Analysis Method [9]

Lin et al. [9] proposed the EMD-based MS analysis method with application to the extraction of the new MS feature information views for HWV samples. The HWV samples, denoted as *hwv*(*t*), were adaptively decomposed into *N* IMFs and one RF using empirical mode decomposition (EMD), as follows:

(1) $$\mathrm{hwv}\left(t\right)=\sum_{i=1}^{N}{\mathrm{IMF}}_{\mathrm{hwvi}}\left(t\right)+\mathrm{rf}\left(t\right),\;\;\;\;$$

where $IMF_{hwvi}\left(t\right)$ and *rf*(*t*) are the *i*-th IMF and RF of the HWV samples, respectively.

The referred total energy of *hwv*(*t*) is provided by

(2) $$E_{\mathrm{hwvref}}=\sum_{i=1}^{N}{\mathrm{IMF}}_{\mathrm{hwvi}}^{2}\left(t\right)+{\mathrm{rf}}^{2}\left(t\right).$$

The energy ratio of the *i*-th IMF to the referred total energy of ***hwv*(*t*)**, $IMFRE_{hwvi}$, is defined [9] as

(3) $${\mathrm{IMFRE}}_{\mathrm{hwvi}}=\frac{{\mathrm{IMF}}_{\mathrm{hwvi}}^{2}\left(t\right)}{E_{\mathrm{hwvref}}}\times 100\%.$$

The energy ratio of the RF to the referred total energy of *hwv*(*t*), $RFRE_{hwv}$, is defined [9] as

(4) $${\mathrm{RFRE}}_{\mathrm{hwv}}=\frac{{\mathrm{rf}}^{2}\left(t\right)}{E_{\mathrm{hwvref}}}\times 100\%,$$

where $z_{hwvi}\left(t\right)$ is expressed as

(5) $$z_{\mathrm{hwvi}}\left(t\right)={\mathrm{IMF}}_{\mathrm{hwvi}}\left(t\right)+\mathrm{jHT}\left\{{\mathrm{IMF}}_{\mathrm{hwvi}}\left(t\right)\right\},\;z_{\mathrm{hwvi}}\left(t\right)=A_{\mathrm{hwvi}}\left(t\right)e^{{j\varphi }_{\mathrm{hwvi}}\left(t\right)}.$$

In Equation (5), $A_{hwvi}\left(t\right)$ and $\varphi_{hwvi}\left(t\right)$ are the amplitude and the phase of $z_{hwvi}\left(t\right)$, respectively, and are provided by

$$A_{hwvi}\left(t\right)=\sqrt{IMF_{hwvi}^{2}\left(t\right)+[HT{\left\{IMF_{hwvi}\left(t\right)\right]}^{2}},\;\varphi_{hwvi}\left(t\right)=\tan^{-1}(\frac{HT\left\{IMF_{hwvi}\left(t\right)\right\}}{IMF_{hwvi}\left(t\right)}).$$

Here, HT{ } is the Hilbert transform.

The *i*-th *IF* of the HWV sample, *IFhwvi*(*t*), is provided by

(6) $${\mathrm{IF}}_{\mathrm{hwvi}}\left(t\right)=\frac{1}{2\pi }\frac{{d\varphi }_{\mathrm{hwvi}}\left(t\right)}{\mathrm{dt}}.$$

The marginal spectrum (MS) of $IMF_{hwvi}\left(t\right)$ in the m–n kHz band [6], i.e., $MSRE_{hwvimn}$ is calculated as

(7) $${\mathrm{MSRE}}_{\mathrm{hwvimn}}=\frac{{\mathrm{IMF}}_{\mathrm{hwvimn}}^{2}\left(t\right)}{E_{\mathrm{hwvref}}}\times 100\%,$$

where $IMF_{hwvimn}^{2}\left(t\right)$ is the energy of $IMF_{hwvi}\left(t\right)$ in the m–n kHz band.

The MS of $RF_{hwv}\left(t\right)$ in the m–n kHz band, $MSRFRE_{hwvmn}$, is calculated as

(8) $${\mathrm{MSRFRE}}_{\mathrm{hwvmn}}=\frac{{\mathrm{rf}}_{\mathrm{hwvmn}}^{2}\left(t\right)}{E_{\mathrm{hwvref}}}\times 100\%,$$

where $rf_{hwvmn}^{2}\left(t\right)$ is the energy of $rf\left(t\right)$ in the m–n kHz band.

## 2. Humpback Whale Vocalizations

**Figure 1:**
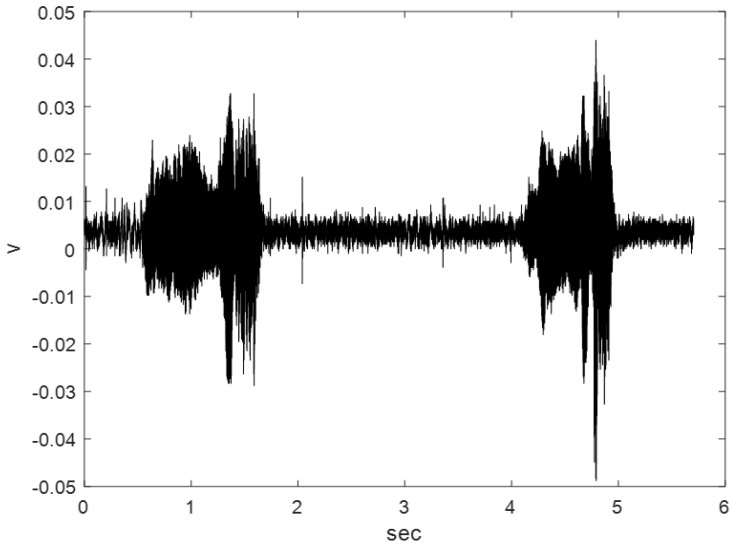
Waveform of the entire HWCV.

An HWV sample (Recording No. 9220100Q) was downloaded from the Watkins Marine Mammal Sound Database [27] (https://cis.whoi.edu/science/B/whalesounds/index.cfm (accessed on 10 August 2023)) with an HWV number. The HWV was recorded in the sea area around the British Virgin Islands (18° N, 64° W) at a water depth of 15 m. Figure 1 shows the full HWV, which is 5.7 s long; the sampling frequency of the vocalization was 14,900 Hz. Thirty-six HWV samples, 17.2 ms in duration, were extracted from the full 9220100Q recording. These samples were categorized into Classes I, II, and III, which contained 15, 5, and 16 samples, respectively. The time-analysis resolution was 17.2 ms so the time-analysis resolution would be high. We evaluated the average energy ratios of the IMF1 and RF to the referred total energy for the Class I samples, the average energy ratios of the IMF1, 2nd IMF (IMF2), and RF to the referred total energy for the Class II samples, and the average energy ratios of the IMF1, 6th IMF (IMF6), and RF to the referred total energy for the Class III samples. All of these energy ratios were larger than 10%. The classification strategies were discussed as follows. The energy ratios of the IMF1 and RF and to the referred total energy were larger than 40% and 20%, respectively, for every Class I sample. The energy ratios of the IMF1 + IMF2 and RF and to the referred total energy of were larger than 55% and 15%, respectively, for every Class II sample. The energy ratios of the IMF1 + IMF6 and RF and the referred total energy were larger than 40% and 55%, respectively, for every Class III sample.

**Figure 2:**
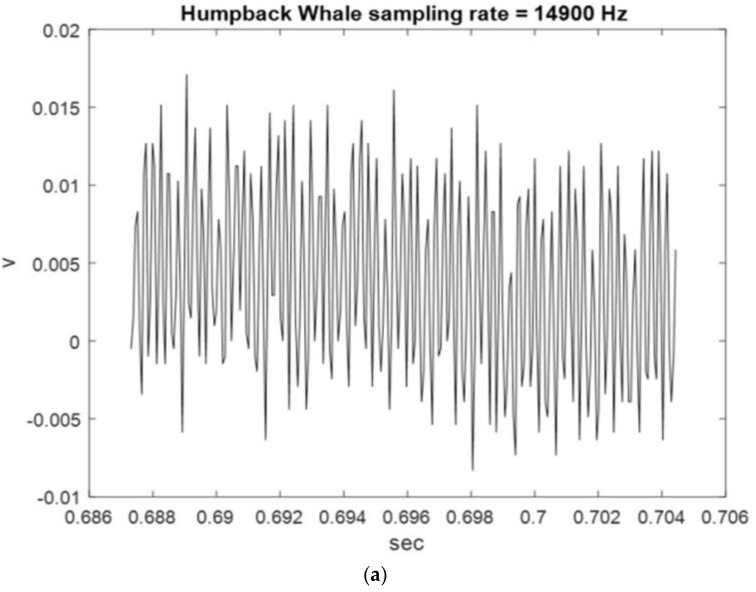

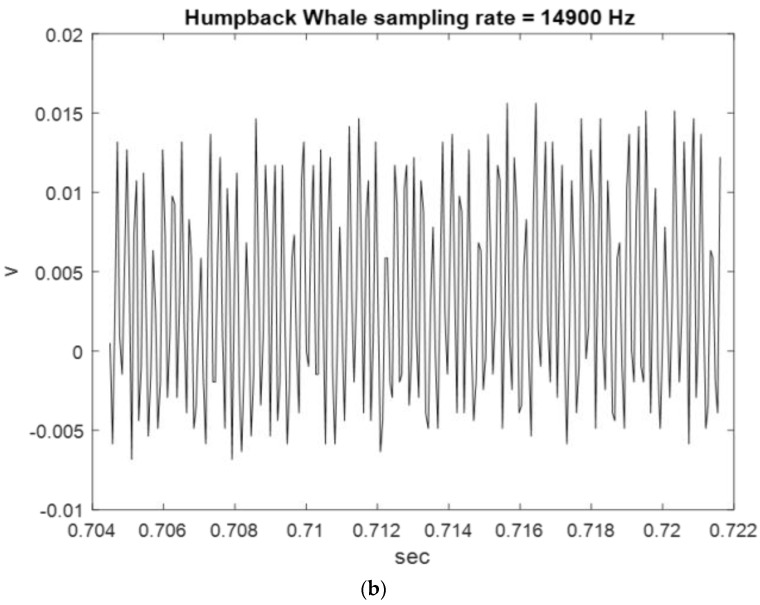
Class I HWV samples: (**a**) sample 1 and (**b**) sample 2.

**Figure 3:**
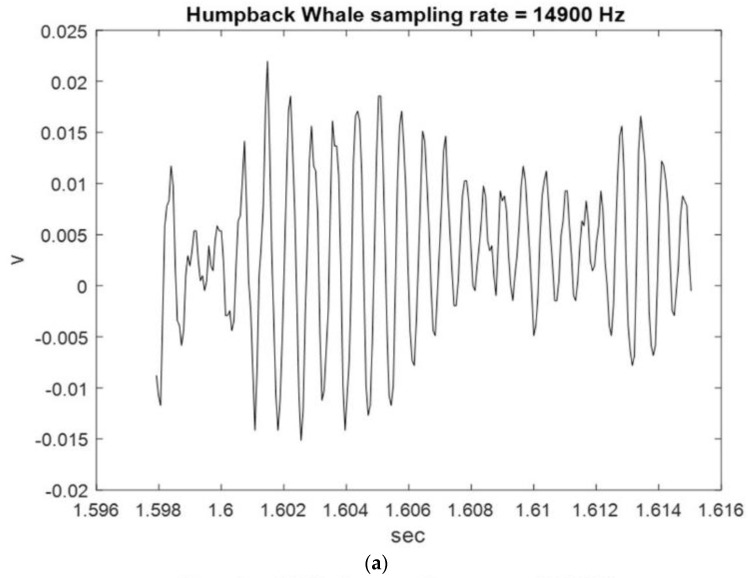

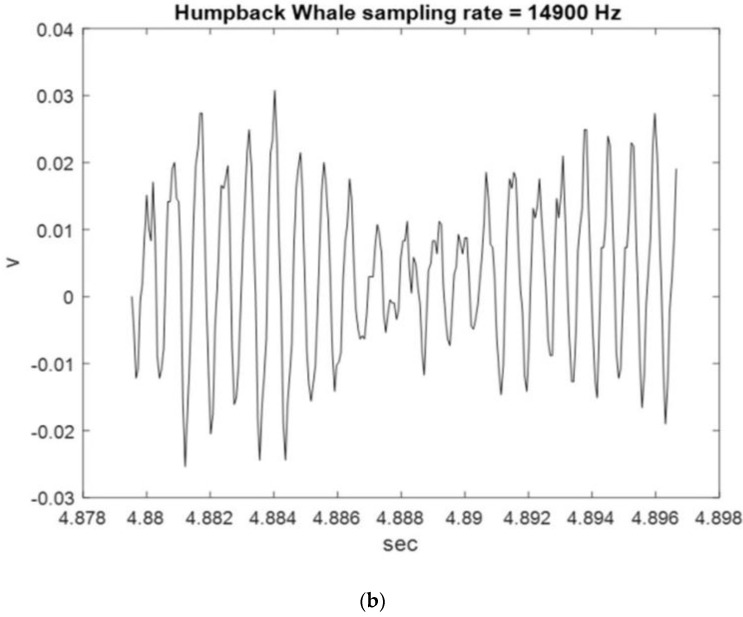
Class II HWV samples: (**a**) sample 1 and (**b**) sample 2.

**Figure 4:**
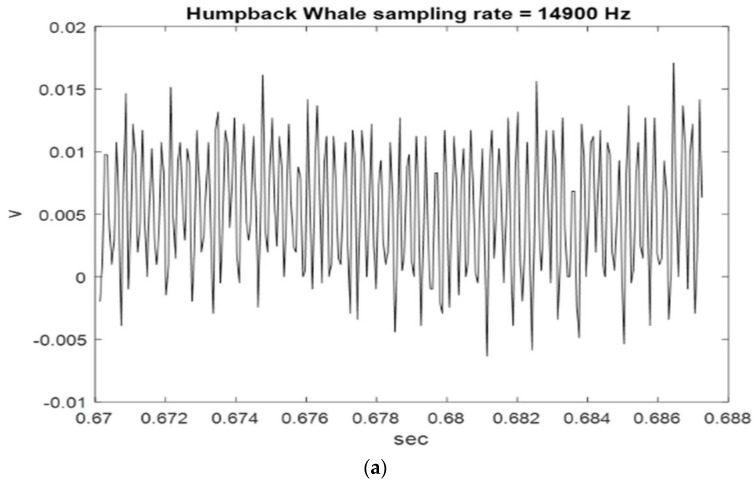

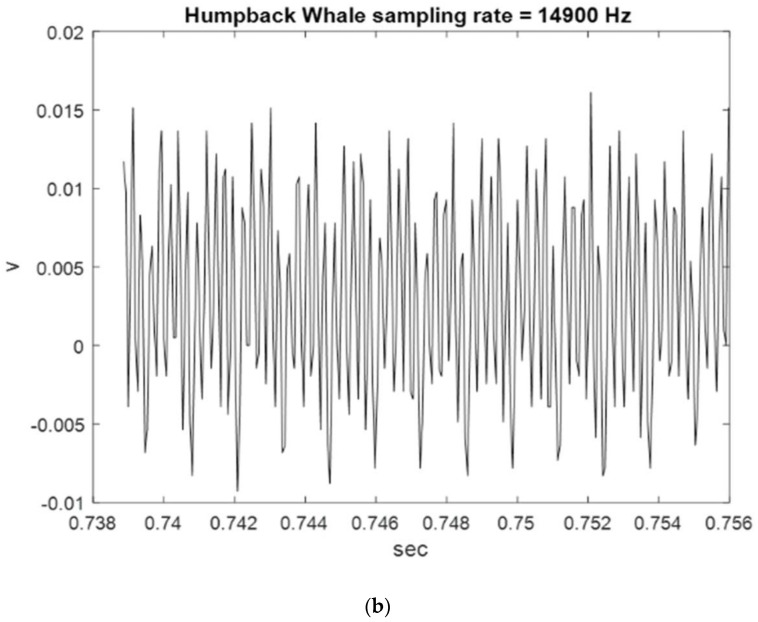
Class III HWV samples: (**a**) sample 1 and (**b**) sample 2.

Figure 2, Figure 3 and Figure 4 show the start and end times of Class I, II, and III HWV samples, which provides a visual sense of these samples. As shown in Figure 2, the start and stop times of samples 1 and 2 for the Class I HWVs, were, respectively, 0.6870–0.7042 s and 0.7043–0.7215 s; they were 1.5977–1.6149 s and 4.8793–4.8965 s for the Class II HWVs (Figure 3) and 0.6700–0.6872 s and 0.7388–0.7560 s for the Class III HWVs (Figure 4). The maximum and minimum amplitudes of the samples for the Class I HWVs were 0.015 V and −0.005 V, respectively. They were 0.010 V and −0.005 V for the Class II HWV samples and 0.015 V and −0.005 V for the Class III HWV samples. Figure 2, Figure 3 and Figure 4 illustrate the wave structure, vision insight of the Class I, II, and III HWV samples, and the amplitude changes with time. Figure 2, Figure 3 and Figure 4 show that Class I, III, and II HWV samples exhibit the fastest, second, and slowest oscillation phenomena (frequencies), respectively, from visual analysis.

## 3. Analysis Results

In this section, the HWV samples were adaptively decomposed into six IMFs and one RF. One original HWV sample in the time domain was expanded to six IMFs and one RF in the time domain; higher resolution TF signal analysis for the HWV samples could be achieved. The wave structure and vision insight of the IMFs and RF for the Class I, II, and III HWV samples were illustrated using the EMD analysis method.

The number of IMFs, for the Class I, II, and III HWV samples were demonstrated. The average instantaneous frequencies (IFs) of IMF1–IMF6 and RF for the Class I, II, and III HWV samples were evaluated. The average energy ratios of the IMF1–IMF6 and RF to the referred total energy and the average energy ratios of IMF1–IMF6 and RF in the several frequency bands were elaborated. The significant and meaningful feature information views, such as the analysis sample duration, number of IMFs, average energy ratios of the significant IMFs and RF to the referred total energy, and average energy ratios of the significant IMFs and RF in the significant frequency bands to the referred total energy for the Class I, II, and III HWV samples were extracted in detail.

### 3.1. Class I HWV Samples

**Figure 5:**
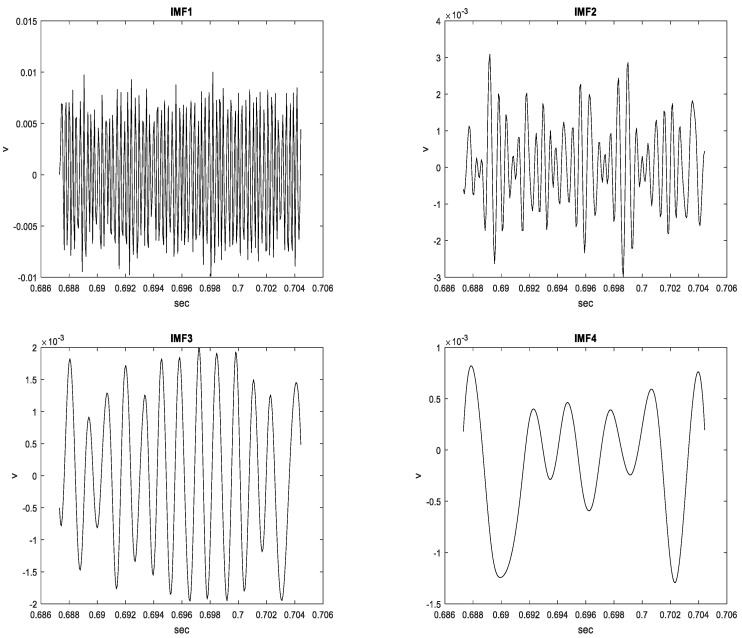

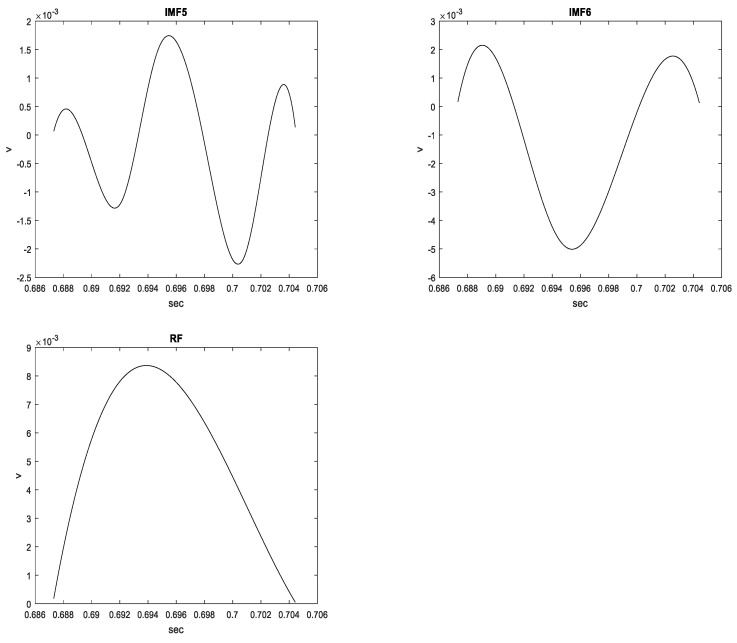
Sample 1 from Class I HWV, adaptively decomposed into six IMFs and one RF.

**Figure 6:**
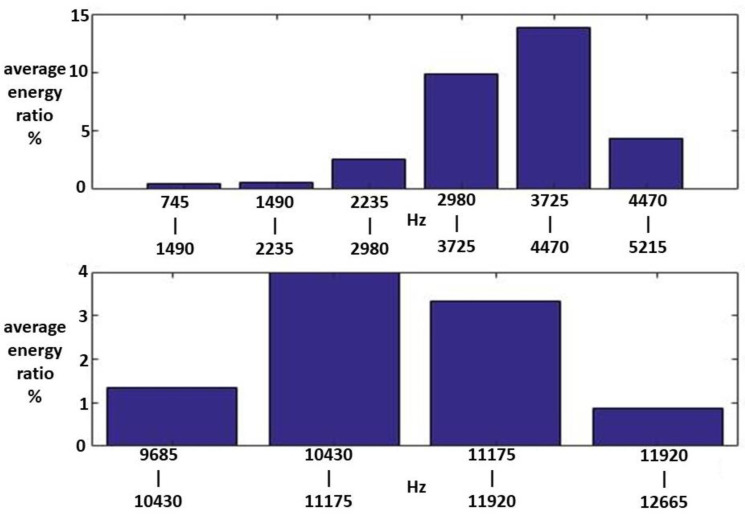
An average MS of IMF1 for the Class I HWV samples.

**Figure 7:**
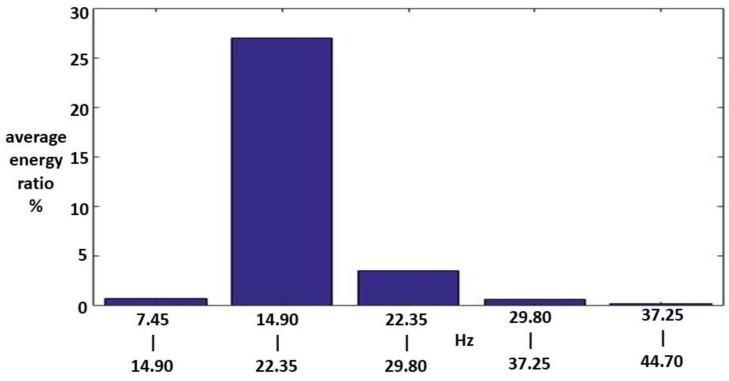
An average MS of RF for the Class I HWV samples.

Fifteen Class I HWV samples were analyzed. Sample 1 from this class was adaptively decomposed into six IMFs and one RF using the EMD method, as illustrated in Figure 5. The number of IMFs depends on the input Class I HWV samples. The average instantaneous frequencies (IFs) of IMF1-IMF6 and RF for Class I HWV sample 1 were 5.633, 3.140, 1.428, 0.582, 0.254, 0.141, and 0.043 kHz, respectively. The average mean IFs of IMF1-IMF6 and RF for Class I HWVs were 5.561, 2.876, 1.313, 0.531, 0.262, 0.137, and 0.040 kHz, respectively. The average energy ratios of the IMF1-IMF6 to the referred total energy of *hwv*(*t*) ($IMFRE_{hwv1}$), $IMFRE_{hwv2}$, $IMFRE_{hwv3}$, $IMFRE_{hwv4}$, $IMFRE_{hwv5}$, and $IMFRE_{hwv6}$, were 46.37%, 4.43%, 4.73%, 1.52%, 2.36%, and 6.37%, respectively, and the average energy ratio of the RF to the referred total energy of ***hwv*(*t*)** ($RFRE_{hwv}$) was 34.21%. Among these results, the energy distributions of IMF1 and RF are significant. Figure 6 and Figure 7 show the average MSs of IMF1 and RF. The average energy ratios of IMF1 in the high-frequency bands of 2.980–3.725, 3.725–4.470, 4.470–5.215, 10.430–11.175, and 11.175–11.920 kHz were 9.825%, 13.790%, 4.398%, 3.977%, and 3.329%, respectively. The average energy ratios of the RF in the low-frequency bands of 14.9–22.35 and 22.35–29.8 Hz were 26.987% and 3.510%, respectively.

### 3.2. Class II HWV Samples

Five Class II HWV samples were analyzed. Sample 1 from this class was adaptively decomposed into six IMFs and one RF using the EMD method. The number of IMFs depended on the input Class II HWV samples. The mean IFs of IMF1–IMF6 and RF for Class II HWV sample 1 were 4.915, 1.445, 0.691, 0.336, 0.135, 0.116, and 0.022 kHz, respectively. The average mean IFs of IMF1-IMF6 and RF for Class II HWVs were 4.218, 1.783, 0.888, 0.481, 0.221, 0.121, and 0.025 kHz, respectively.

The average $IMFRE_{hwv1}$, $IMFRE_{hwv2}$, $IMFRE_{hwv3}$, $IMFRE_{hwv4}$, $IMFRE_{hwv5}$, and $IMFRE_{hwv6}$, were 32.06%, 29.22%, 2.62%, 1.88%, 5.02%, and 6.55%, respectively, and the average $RFRE_{hwv}$ was 22.64%.

**Figure 8:**
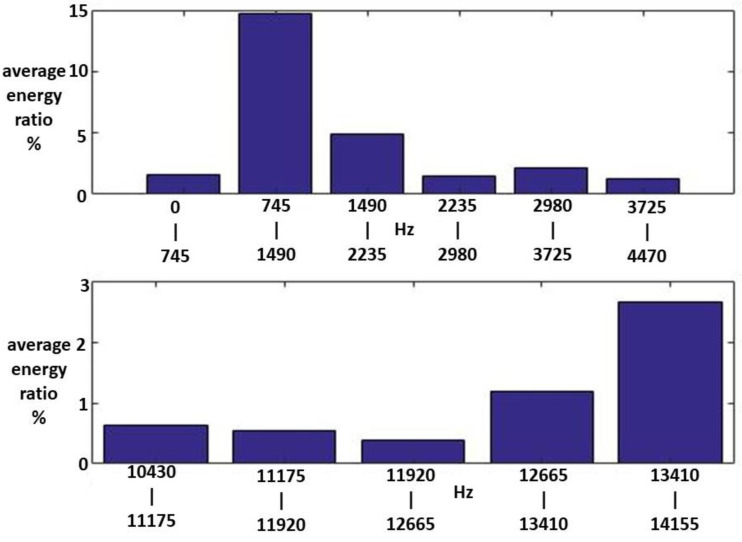
An average MS of IMF1 for the Class II HWV samples.

**Figure 9:**
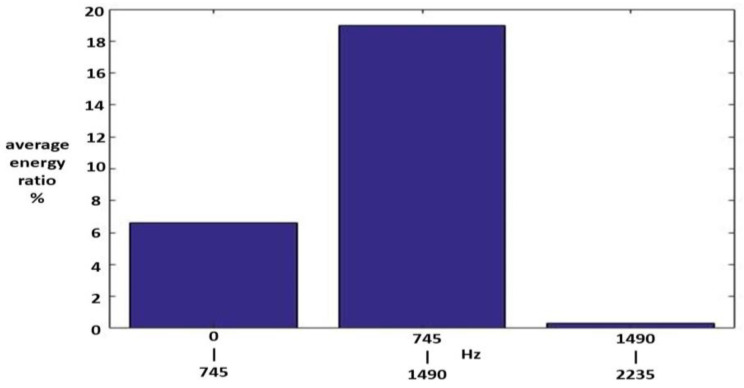
An average MS of IMF2 for the Class II HWV samples.

**Figure 10:**
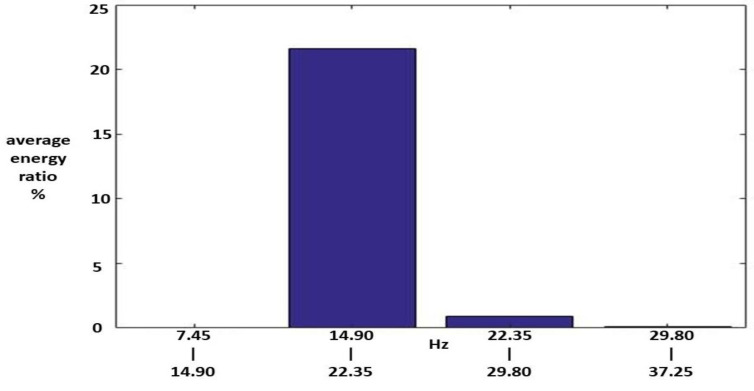
An average MS of RF for the Class II HWV samples.

Among these, the energy distributions of IMF1, IMF2, and the RF were important. Figure 8, Figure 9 and Figure 10 show the average MSs of IMF1, IMF2, and RF. The average energy ratios of IMF1 in the high-frequency bands of 1.490–2.235, 2.235–2.980, 2.980–3.725, 3.725–4.470, 12.665–13.410, and 13.410–14.115 kHz were 14.68%, 4.91%, 1.50%, 2.18%, 1.23%, 1.20%, and 2.66%, respectively. The average energy ratios of IMF2 in the high-frequency bands of 0.745–1.490 and 13.410–14.115 kHz were 18.99% and 2.84%, respectively. The average energy ratio of RF in the low-frequency bands of 14.9–22.35 Hz was 21.63%.

### 3.3. Class III HWV Samples

**Figure 11:**
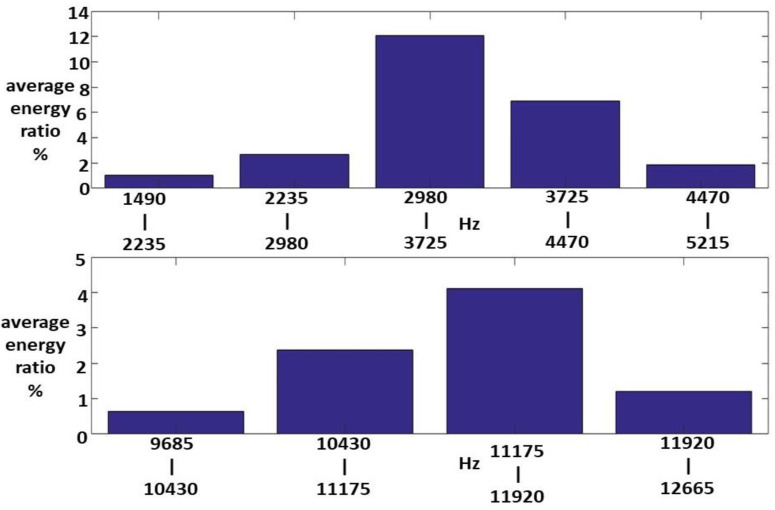
An average MS of IMF1 for the Class III samples.

**Figure 12:**
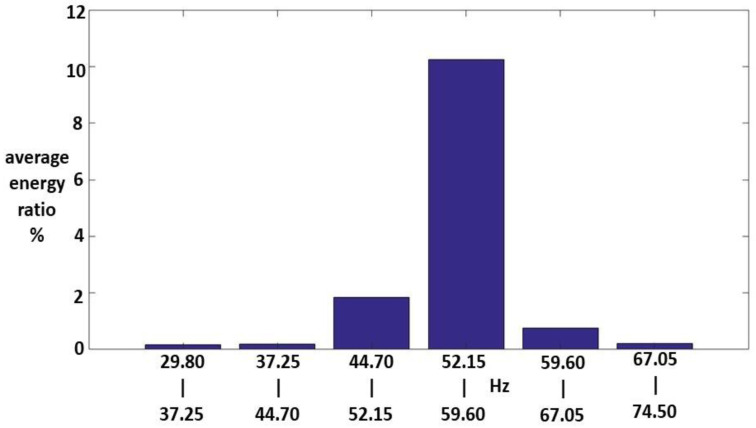
An average MS of IMF6 for the Class III samples.

**Figure 13:**
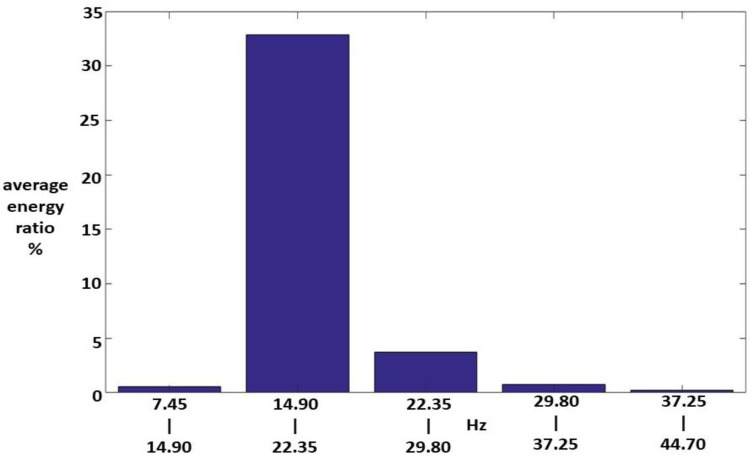
An average MS of RF for the Class III samples.

Sixteen Class III HWV samples were analyzed. Sample 1 from this class was adaptively decomposed into six IMFs and one RF using the EMD method. As for the other classes, the number of IMFs depended on the input Class III HWV samples. The mean IFs of IMF1-IMF6, and RF for Class III HWV sample 1 were 5.720, 2.890, 1.445, 0.330, 0.245, 0.141, and 0.038 kHz, respectively. The average mean IFs of IMF-IMF6, and RF for Class II HWVs were 5.422, 3.153, 1.407, 0.475, 0.242, 0.138, and 0.040 kHz, respectively. The average $IMFRE_{hwv1}$, $IMFRE_{hwv2}$, $IMFRE_{hwv3}$, $IMFRE_{hwv4}$, $IMFRE_{hwv5}$, and $IMFRE_{hwv6}$, were 34.29%, 2.48%, 3.04%, 1.57%, 4.47%, and 15.80%, respectively, and the average $RFRE_{hwv}$ was 38.33%. Among these, the energy distributions of IMF1, IMF6, and the RF were important. Figure 11, Figure 12 and Figure 13 show the average MSs of IMF1, IMF6, and RF. The average energy ratios of IMF1 in the high-frequency bands of 1.490–2.235, 2.235–2.980, 2.980–3.725, 3.725–4.470, 4.470–5.125, 11.175–11.920, and 11.920–12.665 kHz were 1.02%, 2.70%, 12.06%, 6.89%, 1.84%, 4.10%, and 1.20%, respectively. The average energy ratio of IMF6 in the low-frequency bands of 52.15–59.60 Hz was 10.24%. The average energy ratios of RF in the low-frequency bands of 14.9–22.35 and 22.35–29.80 Hz were 32.83% and 3.73%, respectively.

## 4. Discussion

**Figure 14:**
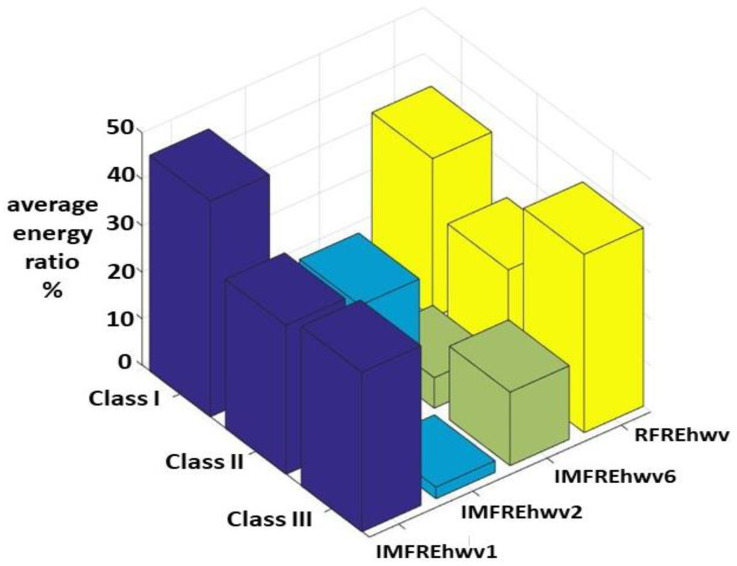
Average values of $IMFRE_{hwvi}$ and $RFRE_{hwv}$ for the Class I, II, and III HWV samples, which were larger than 10%.

Figure 14 shows that the average values of $IMFRE_{hwvi}$ and $RFRE_{hwv}$ for the Class I, II, and III HWV samples were higher than 10%. The average values of $IMFRE_{hwv1}$ for the Class I, II, and III HWV samples were 46.37%, 32.06%, and 34.29%, respectively. The average values of $IMFRE_{hwv2}$ and $IMFRE_{hwv6}$ for the Class II and III HWV samples were 29.22%, and 15.81%, respectively. The average values of $RFRE_{hwv}$ for the Class I, II, and III HWV samples were 34.20%, 22.64%, and 38.32%, respectively. Those results reveal a new characteristic view of significant IMF-based energy distribution.

Table 2 lists the HHT-based feature extraction vocalizations of the HWV, SW clicks [9] samples, and blue whale B call vocalizations (BWBCV). The durations of the Class I, II, and III HWV samples were 17.2, 17.2, and 17.2 ms, respectively; those of the Click I and II SW samples were 10 and 5 ms, respectively; the durations of the Class I and II BWBCV samples were 180 and 180 ms, respectively. The numbers of IMFs for the Class I, II, and III HWV samples were 6, 6, and 6, respectively. Those for the Click I and II SW samples were 7 and 6, respectively, and those for the Class I and II BWBCV samples were 5 and 5, respectively.

The average energy ratios of the IMF1 to the referred total energy for the Class I, II, and III HWV samples were 46.37%, 32.06%, and 34.29%, respectively. The average energy ratios of the IMF1 to the referred total energy for the Click I and II SW samples were 61.50% and 73.33%, respectively. The average energy ratios of the IMF1 to the referred total energy for the Class I and II BWBCV samples were 83.40% and 32.63%, respectively.

The average energy ratios of the IMF2, IMF3, and IMF4 to the referred total energy for the Class II BWBCV samples were 32.63%, 37.00%, 11.95%, and 12.07%, respectively. The highest ratio of the average energy of IMF1 to the referred total energy of the Class I BWBCV samples was 83.40%. Additionally, the highest ratio of the average energy of IMF2 to the referred total energy of the Class II BWBCV samples was 37.00%. Finally, the ratio of the average energy of IMF1 to the referred total energy of the Class II BWBCV samples was the second highest at a ratio of 32.63%. The average energy ratio of the IMF2 to the referred total energy for the Class II HWV samples was 29.22%, and the average energy ratios of the IMF2 to the referred total energy for the Click I and II SW samples were 12.41% and 13.89%, respectively. The average energy ratios of the RF to the referred total energy for the Class I, II, and III HWV samples were 34.21%, 22.64%, and 38.33%, respectively. The energy distributions of IMF1 and IMF2 for the Click I and II SW samples were important.

Table 2 shows that the average values of MS1, MS2, MS6, and MSRF for the Class I, II, and III HWV samples and of MS1, MS2, and MSRF for the Click I and II SW samples in the low and high-frequency bands were all greater than 5%. The average values of MS RF for the Class I, II, and III HWV samples in the 14.90–22.35 Hz band were 26.99%, 21.63%, and 32.83%, respectively, and 7.83% for the Click II SW samples in the 0–1 kHz band. The average value of MS6 for the Class III HWV samples in the 52.15–59.60 Hz band was 10.24%. The average values of MS1 and MS2 for the Class II HWV samples in the 745–1490 Hz band were 14.66% and 18.99%, respectively. The average values of MS1 for the Class I and III HWV samples in the 2980–3725 Hz band were 9.83% and 12.06%, respectively, and 13.79% and 6.89% in the 3725–4470 Hz band, respectively.

The average value of MS1 for the Click I SW samples in the 11–15 kHz band was 30.05%. The average value of MS1 for the Class II SW samples in the 8–15 kHz band was 46.94%, and the average value of MS2 for the Class II SW samples in the 3–7 kHz band was 10.08%. The average value of MS1 for the Class I BWBCV samples in the 34–52 Hz band was 74.18%. The average values of MS1, MS2, MS3, and MS4 were determined for the Class I IBWBCV samples in the 41–52 Hz band with a ratio of 24.08%, in the 10–18 Hz band with a ratio of 28.29%, in the 4–7 Hz band with a ratio of 10.38%, and in the 5–6 Hz band with a ratio of 11.36%. The higher-frequency components of the Class I and II BWBCV samples were 34–52 and 41–52 Hz, respectively. The lower-frequency components of the Class II BWBCV samples were 34–37 Hz.

The higher-frequency components of the Class I, II, and III HWV samples were 3735–4470, 745–1490, and 2980–3725 Hz, respectively, and those of the Click I and II SW samples were 11–15 and 8–15 kHz, respectively. The lower-frequency components of the Class I, II, and III HWV samples were in the range of 14.9–22.35 Hz, and of the Click I and II SW samples were 4–5, and 0–1 kHz, respectively. These results thus reveal the new MS-based energy distribution characteristic views of Class I, II, and III HWV samples.

The average energy ratios of MS1 to the referred total energy in different frequency bands can be added for the proposed Class I HWV samples. The average energy ratios of MS1 in the ranges of 2980–3725 Hz and 3725–4470 Hz to the referred total energy for Class I HWV samples were 9.83% and 13.79%, respectively. The average energy ratio of MS1 in the range of 2980–4470 Hz to the referred total energy for the Class I HWV samples was 23.62%. The average energy ratio of MS1 in the range of 2980–4470 Hz to the referred total energy for Class III HWV samples was 18.95%.

The average energy ratios of MS1 and MS2 to the referred total energy in the same frequency bands can be added for the proposed Class II HWV samples. The average energy ratios of MS1 and MS2 to the referred total energy in the range of 745–1490 Hz were 14.68% and 18.99%, respectively. The average energy ratios of MS1 and MS2 to the referred total energy in the range of 745–1490 Hz for the Class II HWV samples were 33.67%.

Table 1 shows the comparison of the features extracted from HWVs, HWV, HWV songs, and HWV non-song. Using the Fourier analysis method. Frazer et al. [20], Au et al. [21], Kugler et al. [22], Mercado et al. [23], Bilal et al. [24], and Angela et al. [25] demonstrated the important frequency bands of HWV to be in the ranges of 20–8000 Hz, >21 kHz, 0–1500 Hz, <400 Hz and 3000–8000 Hz, 8–8000 Hz, and 9–6000 Hz, respectively. Table 2 lists the HHT-based feature extraction vocalizations of HWV. The important high-frequency bands of the proposed HWV Class I, II, and III features were in the ranges of 2980–4470 Hz, 745–1490 Hz, and 2980–4470 Hz, respectively. The important low-frequency bands of the proposed HWV Class III features were in the range of 52.15–59.60 Hz. The important low-frequency bands of the proposed HWV Class I, II, and III features were in the ranges of 14.90–22.35 Hz, 14.90–22.35 Hz, and 14.90–22.35 Hz, respectively. The results of this study provide a better understanding, high resolution, and new innovative views on the information obtained from the MS features of the HWV signals.

## 5. Conclusions

In the paper, 36 HWV samples were classified into Classes I, II, and III, which consisted of 15, 5, and 16 samples, respectively. These samples were decomposed into six IMFs and one RF using the EMD method. The first sample of Class I was illustrated. The average values of $IMFRE_{hwv1}$ and $RFRE_{hwv}$ for the Class I samples, $IMFRE_{hwv1}$, $IMFRE_{hwv2}$, and $RFRE_{hwv}$ for the Class II samples, and $IMFRE_{hwv1}$, $IMFRE_{hwv6}$ and $RFRE_{hwv}$ for the Class III samples were all greater than 10%.

The average important energy ratios of IMF1 to the referred total energy for the Class I, II, and III HWV samples were 46.37%, 32.06%, and 34.29%, respectively. The average important energy ratios of RF to the referred total energy for the Class I, II, and III HWV samples were 34.21%, 22.64%, and 38.33%, respectively. The average important energy ratios of MS1 in the high-frequency bands of 2980–3725 and 3725–4470 Hz to the referred total energy for the Class I HWV samples were 9.83% and 13.79%, respectively. The average important energy ratio of the MS1 in the high-frequency band of 745–1490 Hz to the referred total energy for the Class II HWV samples was 14.68%.

The average important energy ratios of the MS1 in high-frequency bands of 2980–3725 and 3725–4470 Hz to the referred total energy for the Class III HWV samples were 12.06% and 6.89%, respectively. The average important energy ratio of the MS2 in the high-frequency band of 745–1490 Hz to the referred total energy for the Class II HWV samples was 18.99%. The average important energy ratio of the MS6 in the low-frequency band of 52.15–59.60 Hz to the referred total energy for the Class II HWV samples was 10.27%. The average important energy ratios of the MS RF in the low-frequency band of 14.90–22.35 Hz to the referred total energy for the Class I, II, and III HWV samples were 26.99%, 21.63%, and 32.83%, respectively.

The MS characteristics of Class I, II, and III samples in the high and low-frequency bands were revealed. The high time and frequency analytical resolutions of the proposed HHT-based analysis method for HWV samples were 17.2 ms and 7.45 Hz, respectively. High TF analytical resolutions of HWV samples were achieved. The proposed MS-based analytical method for HWV samples is easy to implement using software and hardware, and a short analytical time can be achieved. The results of this paper provide a better understanding of the IMF and MS energy distribution characteristics of HWV samples when HHT-TF analytical methods are used. EMD-based analysis results show that the analysis sample duration, number of IMFs, significant and meaningful IMFs, and significant and meaningful RF, MS1, MS2, MS6, and MS RF new feature information views were revealed.

## Figures and Tables

**Table 1:** Comparison of features extracted from (a) BFW LFS, BMSWV DP, BBW ES, KW FMTUC, and BOWV; (b) KW DSCV, BW song, and HWVs; (c) HWV, HWV songs, and HWV non-song.

(a)					
	Whitlow et al. [12]	Goold et al. [13]	Keating et al. [14]	Houser et al. [15]	Madhusudhana et al. [16]
Whale species	BFW LFS	BMSWV DP	BBW ES	KW FMTUC	BOWV
Analysis method	PAMS	Fourier	Fourier	Fourier	Fourier
Important band	10–20 Hz	400–2000 Hz	Center frequency 39.5 kH 10 dB bandwidth 9.3 kHz	10–130 kHz	15–62 Hz
**(b)**					
	**Reyes** **et al.** [18]	**Malige** **et al.** [19]	**Frazer** **et al.** [20]	**Au** **et al.** [21]	**Kugler** **et al.** [22]
Whale species	KW DSCV	BW song	HWV	HWV	HWV
Analysis method	Fourier	Fourier	Fourier	Fourier	Fourier
Important band	15.7–21.6 kHz 10 dB bandwidth 5.9 kHz	Peak frequency 32 Hz		>21 kHz	0–1500 Hz
**(c)**					
	**Mercado** **et al.** [23]	**Bilal** **et al.** [24]	**Angela** **et al.** [25]		
Whale species	HWV	HWV song	HWV Non-song		
Analysis method	Fourier	Fourier	Fourier		
Important band	<400 Hz 3–8 kHz	Hz	9–6000 Hz		

**Table 2:** Comparison of (a) HHT-based features extracted from HWV, SW click samples, and blue whale B call vocalizations; (b) HHT-based features extracted from HWV, SW click samples, and blue B call whale vocalizations.

(a)				
	Proposed Features Class I	Proposed Features Class II	Proposed Features Class III	Lin et al. [9] Click I
Whale species	HWV	HWV	HWV	SW
Analysis sample duration	17.2 ms	17.2 ms	17.2 ms	10 ms
Number of IMFs	6	6	6	7
Important IMFs	IMF1 (46.37%)	IMF1 (32.06%) IMF2 (29.22%)	IMF1 (34.29%) IMF6 (15.80%)	IMF1 (61.50%) IMF2 (12.41%)
Important RF	34.21%	22.64%	38.33%	-
MS1	2980–3725 Hz (9.825%) 3725–4470 Hz (13.79%)	745–1490 Hz (14.675%)	2980–3725 Hz (12.064%) 3725–4470 Hz (6.885%)	11–15 kHz (30.05%)
MS2	-	745–1490 Hz (18.990%)	-	4–5 kHz (1.20%) 6–7 kHz (1.04%)
MS3	-	-	-	-
MS4	-	-	-	-
MS6	-	-	52.15–59.60 Hz (10.237%)	-
MS RF	14.9–22.35 Hz (26.987%)	14.9–22.35 Hz (21.633%)	14.9–22.35 Hz (32.828%)	-
Application	Features extraction	Features extraction	Features extraction	Features extraction
**(b)**				
	**Lin et al.** [9] **Click II**	**Wen et al.** [10] **Class I**	**Wen et al.** [10] **Class II**	**Adam** [6]
Whale species	SW	BWBCV	BWBCV	KW
Analysis sample duration	5 ms	180 ms	180 ms	650 ms
Number of IMFs	6	5	5	13
Important IMFs	IMF1 (73.33%) IMF2 (13.89%)	IMF1 (83.40%)	IMF1 (32.63%) IMF2 (37.00%) IMF3 (11.95%) IMF4 (12.07%)	-
Important RF	-	-	-	-
MS1	-	34–52 Hz (74.18%)	41–52 Hz (24.08%)	-
MS2	8–15 kHz (46.94%)	-	10–18 Hz (28.29%)	-
MS3	3–7 kHz (10.08%)	-	4–7 Hz (10.38%)	-
MS4	-	-	5–6 Hz (11.36%)	-
MS6	-	-	-	-
MS RF	0–1 kHz (7.83%)	-	-	-
Application	Features extraction	Features extraction	Features extraction	Denoise and Features extraction

## Data Availability

Not applicable.

## Abbreviations

BBW	Blainville’s beaked whales
BFW	blue and fin whales
BMSWV	bull male sperm whale vocalizations
BOWV	Balaenoptera omurai whale vocalizations
BW	blue whale
DP	distinctive peak
DSCV	down-swept contour vocalizations
ES	echolocation signals
EMD	empirical mode decomposition
FMTUC	frequency-modulated tonal up-chirp
HHT	Hilbert–Huang transformation
HT	Hilbert transform
HW	humpback whale
HWV	humpback whale vocalization
IMF	intrinsic mode function
IF	instantaneous frequency
KW	killer whales
LFS	low-frequency sounds
MS	marginal spectrum
PAMS	passive acoustic monitoring system
RF	residual function
R-type	resident-type
SW	sperm whale
TF	time–frequency
